# Molecular Epidemiological Survey of Canine Parvovirus Circulating in China from 2014 to 2019

**DOI:** 10.3390/pathogens10050588

**Published:** 2021-05-11

**Authors:** Bixia Chen, Xiaohui Zhang, Jie Zhu, Lijing Liao, Endong Bao

**Affiliations:** 1 Veterinary Pathology Laboratory, College of Veterinary Medicine, Nanjing Agricultural University, Nanjing 210095, China; 2019807112@njau.edu.cn (B.C.); zhangxh@njau.edu.cn (X.Z.); 2019107001@njau.edu.cn (J.Z.); 2 Pet Department, Ringpu Biotechnology Company, Tianjin 300308, China; see026@outlook.com

**Keywords:** canine parvovirus, feline parvovirus, epidemiological surveillance, amino acid substitutions

## Abstract

The global distribution of canine parvovirus (CPV-2) derived from a closely related carnivore parvovirus poses a considerable threat to the dog population. The virus is continuously undergoing genetic evolution, giving rise to several variants. To investigate the prevalence of Chinese CPV-2 strains in recent years, a total of 30 CPV-2 strains were collected from 2018 to 2021 and the VP2 gene was sequenced and analyzed. Two variants, new CPV-2a (297Ala, 426Asn) and CPV-2c (426Glu), were identified. In contrast to previous reports, the CPV-2c variant has gained an epidemiological advantage over the new CPV-2a variant in China. To compensate for the relatively small sample size, 683 Chinese CPV-2 strains identified between 2014 and 2019 were retrieved from the GenBank database and previous publications, and analyses of these strains further supported our findings, which should be considered since the CPV-2c variant has been frequently associated with immune failure in adult dogs. VP2 protein sequence analysis revealed several amino acid substitutions, including Ala5Gly, Pro13Ser, Phe267Tyr, Tyr324Ile, Gln370Arg, Thr440Ala, and Lys570Arg. Phylogenetic analysis of full-length VP2 gene indicated a close relationship between Chinese strains and other Asian strains, suggesting mutual transmission between Asian countries. Furthermore, intercontinental transmission is a cause for concern. Surprisingly, two feline panleukopenia virus (FPV) strains with the Ile101Thr mutation in the VP2 protein were identified in canine fecal samples; FPV has been considered incapable of infecting dogs. This study clarified the epidemic characteristics of Chinese CPV-2 strains detected between 2014 and 2019, offering a reference for epidemic control. In addition, the detection of FPV in canine samples may provide information for future studies on the evolution of carnivore parvoviruses.

## 1. Introduction

Canine parvovirus (CPV-2), a causative agent of hemorrhagic gastroenteritis and myocarditis in canids, belongs to the *Parvoviridae* family, *Parvovirinae* subfamily, and *Protoparvovirus* genus [1]. The clinical symptoms include fever, leukopenia, diarrhea, dehydration, and anorexia with 100% morbidity and a mortality of 10% in adult dogs and 91% in puppies [2].

CPV-2 is a naked, icosahedral, linearized, and single-stranded DNA virus [3]. The capsid consists of 60 subunits. Each subunit has the same eight-stranded antiparallel β-barrel motif with large insertions between the strands of the β-barrel [4]. The features of the capsid include spikes in threefold axes, a canyon-like depression surrounding each fivefold axis, and a dimple-like depression in twofold axes [5]. The full genome is 5323 bp, containing two open reading frames (ORFs) [6]. One ORF is located at the 3′ end and encodes nonstructural proteins called NS1 and NS2. NS1 is necessary for duplication [7], while the function of NS2 is unclear and does not seem to be required for efficient replication and assembly [8]. Another ORF is located at the 5′ end and encodes structural proteins, including VP1 and VP2, through alternative splicing of the same Mrna [9]. The VP1 protein encompasses the complete sequence of the VP2 protein and has a unique 143-residue N-terminal sequence that is required for successful infection [10]. The VP2 protein is preferentially mutated and a key molecule for determining host range, antigenic properties, and receptor binding. The antigens or subtypes of CPV-2 can be identified by certain residues within the VP2 protein. VP3 is derived from the VP2 protein by host proteolytic cleavage as is presented only on complete (DNA-containing) virions [11].

CPV-2 is regarded as a host-range variant derived from feline parvovirus (FPV) or an FPV-like virus in wild carnivores that gained the ability to bind canine transferrin receptors (TfRs), thus allowing for the infection of dogs but failing to replicate in feline hosts [12,13,14]. CPV-2 was first reported in 1978 in the United States; however, serological tests indicated that dogs in Europe or Eurasia were widely infected between 1974 and 1976 [15]. At least six amino acid differences between CPV-2 and FPV have been found. VP2 amino acid residues at positions 93 and 323 determine the canine host range [16]. Within a few years of its discovery, CPV-2 variants were found, replacing original CPV-2. The first CPV-2 variant, termed CPV-2a, emerged in 1979. The single mutation of VP2 residue 300, from Asp to Gly, is a key determinant of the feline infection [17]. In 1984 and 2001, another two variants, CPV-2b and CPV-2c, were detected in the United States and Italy, respectively. The antigenic differences among these three variants were based on the amino acid 426 of the VP2 protein (Asn in CPV-2a, Asp in CPV-2b and Glu in CPV-2c) [9].

Additional variants, new CPV-2a and new CPV-2b, were discovered in 1990. These variants differ from CPV-2a/b only at residue 297 of the VP2 protein (with the variants having a Ser not a Ala) which does not change the antigenic properties of the variants or even their proximity to epitope B [18], which is currently detected in most recent CPV-2 strains [19]. Two other variants, CPV-2c(a) and CPV-2c(b), were isolated from Vietnamese leopard cats in 1997; they have a substitution at residue 300 (Gly to Asp) and loss of the canine host range [20]. The few amino acid differences in the VP2 protein between the FPV, CPV-2 and CPV-2 variants appear to have modified important biological properties, such as antigenic properties, host ranges, interactions with TfR, and virulence [3].

In China, the first CPV-2 strain was detected in 1982 [21]. CPV-2 prevailed during the early 1980s and was gradually replaced by CPV-2a after 1986 [22]. In the 1990s, most of the CPV-2 variants detected were new CPV-2a/2b variants, which seemed to completely replace the original CPV-2a/2b strains. New CPV-2a has been the dominant genotype since the 1990s [23,24]. The CPV-2c variant was initially detected in 2009 and has shown a continuous uptrend in infections since 2010 [23,25]. In this study, we collected samples in the Tianjin area, a city that has not been previously investigated, and other cities between 2018 and 2021. To further study the prevalence of CPV-2 in China, we retrieved CPV-2 sequence data with clear backgrounds collected in China and entered in the GenBank database and data from related papers published between 2014 and 2019 and examined these data in detail.

## 2. Results

### 2.1. Virus Isolation and PCR Results

Twenty-four viral strains were successfully isolated from collected fecal samples and showed positive PCR results (results not shown). The DNA of 6 strains was directly extracted from fecal samples and then sequenced.

### 2.2. Amplification of the VP2 Gene and Genotype Analysis

Full-length VP2 nucleotide sequences were amplified from 30 samples, which were 2293 bp in length. After alignment with reference strains, extra sequence length was cut off, reducing the sequence to 1755 bp, which was the full VP2 gene. Table 1 provides background information on the CPV-2 strains isolated in this study.

The results revealed the cocirculation of new CPV-2a (297Ala, 426Asn) and CPV-2c (426Glu) strains in China. No CPV-2, CPV-2a (297Ser, 426Asn), CPV-2b (297Ser, 426Asp), or new CPV-2b (297Ala, 426Asp) variants were detected. CPV-2c was the predominant variant detected in 21 samples (70%), while new CPV-2a variants were only detected in 7 samples (23%). Notably, two FPVs were identified in canine samples. FPV and CPV-2 share 99% nucleotide identity, therefore enabling a single pair of primers to detect both FPV and CPV-2. FPV was characterized by 80Lys, 93Lys, 103Val, 323Asp, 564Asn, 568Ala [26]. Thus, these two strains were identified as FPV. It was reported that FPV strains can replicate only in feline cells [27]; however, this study found two exceptions of FPV replicating in canine cells.

### 2.3. Sequence Analysis

Twenty-two nucleotide mutations resulting in nonsynonymous substitutions in the VP2 protein were identified (Table 2). All CPV-2 strains presented residue mutations at Phe267Tyr and Tyr324Ile. All new CPV-2a strains carried a specific mutation, Thr440Ala, and most CPV-2c strains exhibited the unique mutations Ala5Gly and Gln370Arg, while one new CPV-2a strain also harbored a Ala5Gly mutation. Mutation at residue Pro13Ser was evident in two CPV-2c strains and one new CPV-2a strain. Another mutation was identified as Lys570Arg in one CPV-2c strain. Mutation Ile101Thr was observed in two FPV strains, and Pro238Gln was observed in one FPV strain.

### 2.4. Phylogenetic Analysis

Considering the evaluation of MEGA-X, an ML phylogenetic tree based on the full VP2 gene was constructed with a Tamura 3-parameter model with gamma distribution and five rate categories (T92+G+I).

**Figure 1:**
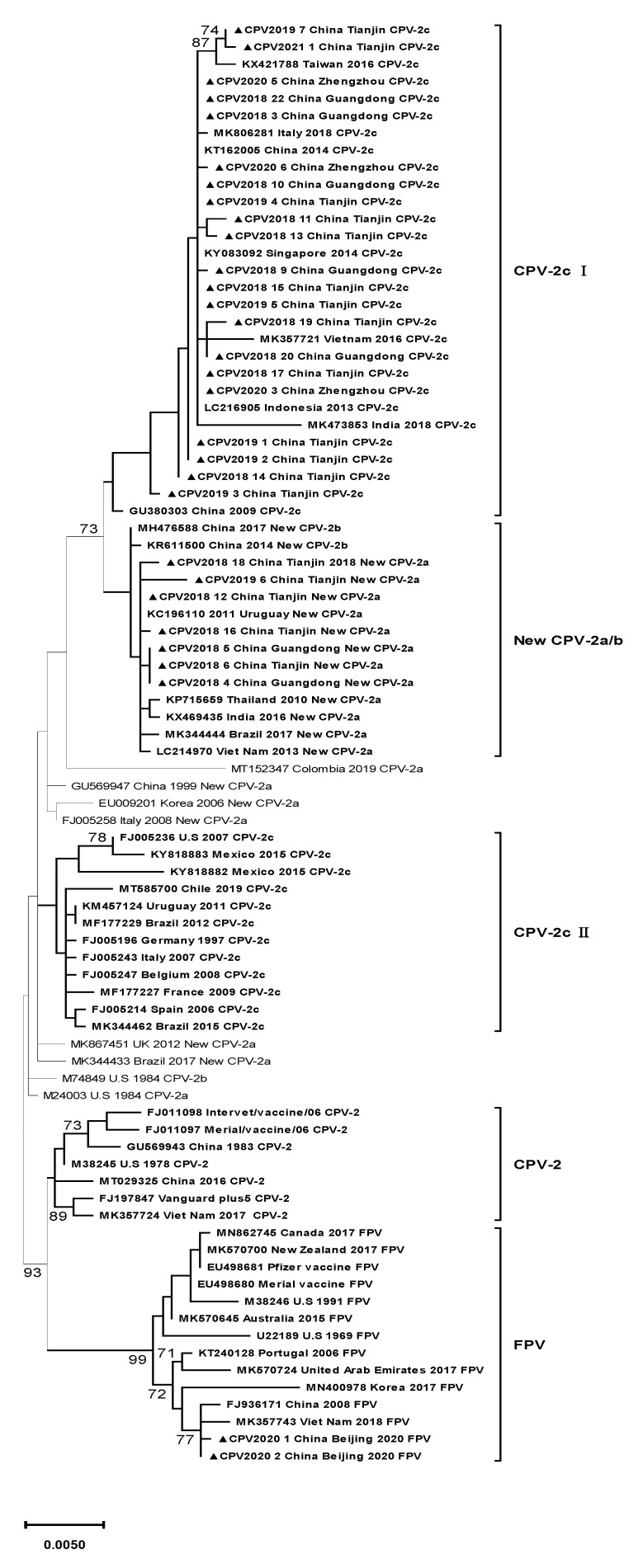
Maximum likelihood phylogenetic tree based on the full VP2 gene of canine parvovirus (CPV-2). Findings with bootstrap values less than 70% are not shown on the branches. Strains analyzed in this study are marked with ▲. Reference strains are exhibited in the following order: GenBank accession number, country where virus was isolated, isolation time and genotype. The primary residue mutations in every clade are noted within a hollow rectangle.

According to the phylogenetic tree, the 30 strains were clustered into three clades (Figure 1). Clade CPV-2c Ⅰ included all the CPV-2c variants in this study, Asian CPV-2c strains, and one Italian CPV-2c strain. This clade was distinguished from clade CPV-2c Ⅱ, which contained CPV-2c strains circulating in European and American countries. The CPV-2c strains belonging to this clade were characterized by four mutations: Ala5Gly, Phe267Tyr, Tyr324Ile, and Gln370Arg. Seven new CPV-2a strains in this study were classified into another clade, New CPV-2a/b, containing Asian new CPV-2a/b strains and two American new CPV-2a strains. All the strains in this clade shared three mutations, Phe267Tyr, Tyr324Ile, and Thr440Ala, clustering away from other new CPV-2a variants with different mutations. Two FPV strains were located in clade FPV, which included all the FPV strains.

### 2.5. Temporal Distribution Analysis

A total of 683 Chinese CPV-2 records were retrieved from the GenBank database and related publications. The details are presented in Supplementary Materials (Table S1). Additionally, 24 strains (collected from 2018 to 2019) identified in this study were included in the analyses.

**Figure 2:**
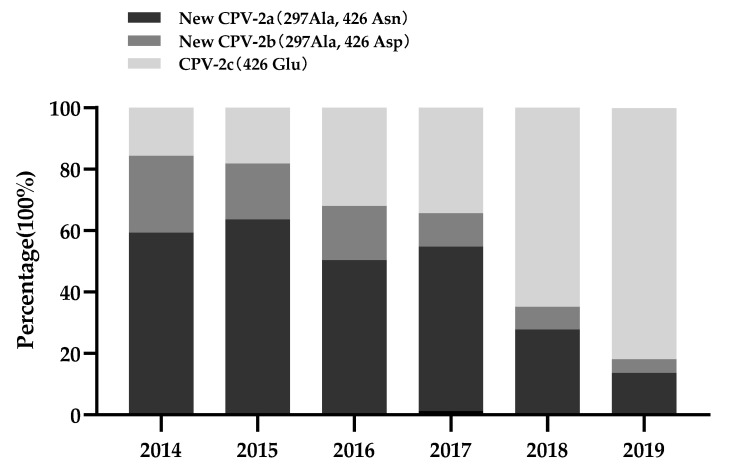
Temporal distribution of CPV-2 in China from 2014 to 2019.

The temporal distribution analysis is shown in Figure 2. The detection rate of new CPV-2a (297Ala, 426Asn) has declined since 2016, and new CPV-2b (297Ala, 426Asp) showed the same decreasing tendency. In contrast, the detection rate of CPV-2c (426Glu) has been consistently increasing since 2014, thereby replacing new CPV-2a as the predominant variant in China after 2018. However, this temporal distribution is based on incomplete GenBank submissions and related publications. Thus, it does not reflect the actual prevalence of CPV-2 in China.

## 3. Discussion

According to reports before 2015, CPV-2c (426Glu) was rarely detected in China [24,28,29,30,31]. After 2015, although its number increased, new CPV-2a (297Ala, 426Asn) constituted the highest proportion of cases in China according to most reports [32,33,34,35,36]. The results from this study are different than those of previous reports. By collecting available data on Chinese CPV-2 strains from the GenBank database and related publications, we further proved our finding that CPV-2c dramatically increased in China and ultimately replaced the new CPV-2a variant as the predominant strain in most cities of China. However, since many cities were not investigated, these results are not conclusive. However, this tendency should be considered since CPV-2c variants have been frequently associated with CPV-2 outbreaks in vaccinated dogs [3,26,37]. To date, there is no consensus about the effectiveness of prototype CPV-2-based vaccines against heterologous CPV-2 variants [38]. However, the continuous and dynamic evolution of CPV-2c may pose a new challenge to the effectiveness of the vaccines currently in use.

In the past, CPV-2c was found mainly in South American and European countries, and it was rarely detected in Asia, where a higher percentage of CPV-2a/2b variants prevailed [9,26]. However, in recent years, the frequency of CPV-2c detection has increased in Asia [39]. The results of this study were consistent with recent reports of Asian countries, such as Taiwan [40,41], Laos [42], Vietnam [43,44], Thailand [45,46], and Korea [39]. These reports either revealed the higher detection rate of CPV-2c or indicated that CPV-2c has become the predominant variant in recent years. Notably, these reports have indicated that the isolated strains share features similar to those of the Asian strains, which seems to indicate mutual transmission of CPV-2 between neighboring Asian countries. We have observed synchronous changes in CPV-2c in some Asian countries since 2015; thus, it is possible that CPV-2c variants have continued to evolve in Asia and gained a stronger epidemiological advantage over other mutants.

In this study, several amino acid mutations in the VP2 protein were found and some of them have been discussed extensively before. Mutations at Phe267Tyr, Tyr324Ile, and Gln370Arg were the characteristic of CPV-2 strains of Asian origin [39]. The Ala5Gly mutation was previously observed only in Asian strains; however, it has also recently been found in CPV-2a/b variants in Australia [47] and Italy [48]. This mutation may not be of unitary origin but evolved independently due to the increasingly selective pressure at this site, as indicated by this mutation not being evident in the same variants. The Thr440Ala mutation was first identified in 1993 and became prevalent after 2005 [49]. Residue 440 is found in the threefold axis, a site containing two major epitope [50]. Thus, mutations in this site may have antigenic significance and affect the host immune response. This mutation was estimated to have multiple evolutionary origins [51,52].

Another substitution, Pro13Ser, was found in one new CPV-2a strain and two CPV-2c strains. This mutation was identified in Uruguayan, Vietnamese, and Japanese dogs, European cats [52], and Chinese raccoon dogs [53]. In Italy, a mutation of residue 13 was observed in CPV-2b strains, but the substitution involved Ala not Ser [54]. It was estimated that this mutation may not be antigenically significant since residue 13 was not exposed on the virus surface [53].

The last mutation found to date was observed in residue 570, in which a Lys residue is replaced with an Arg residue. This mutation has not been reported in China. However, in two Australian strains and one vaccine strain, the Lys residue is replaced with a Glu residue [47]. Residue 570 lies on the capsid surface and is close to residue 300, a residue that has the greatest variability and determines the cross-species transfer of viruses between carnivores [55]. Thus, alteration of residue 570 may also affect the host TfR binding ability.

Considering the phylogenetic tree inferred from VP2 sequences, all the CPV-2c strains in this study, along with other newly sequenced Chinese CPV-2c strains, were clustered in the same clade, indicating an intimate relationship between Chinese CPV-2c strains, which suggests mutual transmission between different cities in China. Other Asian strains were in the same clade as the Chinese strains, revealing the possible introduction of variants from neighboring Asian countries into China. Notably, one Italian strain was also included in this clade; however, this strain was reportedly of Asian origin [48]. In recent years, several studies have detected CPV-2 strains originating from Asia in Oceanian, African, American, and European countries [47,48,53,56,57,58]. Therefore, stricter border controls may be needed as a preventive measure. All the strains located in this clade carried four important substitutions: Ala5Gly, Phe267Tyr, Tyr324Ile, and Gln370Arg. Clade CPV-2c Ⅱ contained CPV-2c strains retaining initial amino acids at these sites and circulated in American and European countries, suggesting the geographical isolation of CPV-2c variants in Asian countries.

Compared to CPV-2c variants, the molecular evolution of new CPV-2a strains was more complicated due to its genetic diversity. New CPV-2a variants were marked by residue 297Ala and 426 Asn. However, in Colombia, Brazil, Uruguay, and Argentina [56], residue 297 may be substituted with Asn. Thus, such categorization can be confusing. All the new CPV-2a strains isolated in this study were located in the same clade, characterized by the mutations Phe267Tyr, Tyr324Ile, and Thr440Ala, while some new CPV-2a strains were not included in this clade. The three mutations resulted in a distant phylogenetic relationship between new CPV-2a strains in clade new CPV-2a/b and other new CPV-2a strains, which highlights the importance of these mutations. All the FPV strains were located in the FPV clade. Compared with CPV-2, FPV strains were highly conserved and exhibited a closer relationship with Asian strains. However, the analysis of international distribution of viruses should take sampling biases into account.

Remarkably, two FPV strains were detected in canine samples, and both harbored the Ile101Thr mutation, which is CPV-2 mutant-specific. Most FPV strains detected recently had the same mutation [59,60]. These two FPVs were closely related and may from the same outbreak. This finding is unexpected since FPV is unable to replicate in the small intestine or mesenteric lymph nodes of dogs without being shed in the feces [61]. Similar findings have been reported for Pakistani and Thai strains. This phenomenon may suggest the dynamic evolution of carnivore parvovirus or the existence of a recombinant virus of CPV and FPV [45,62]. However, this outcome may have been the results of a mistake made by a veterinary assistant, mislabeling feline samples as canine samples; however, reports in recent years seem to disprove this postulation. Additionally, as Nisar Ahmed suggested, the occurrence of FPV in dogs is possibly due to the coprophagous behavior of dogs [62]. Further investigations are needed to explain this phenomenon.

## 4. Materials and Methods

### 4.1. Sample Collection

Thirty samples were collected from small animal clinics between 2018 and 2021. When a dog tested positive using the Rapid CPV/CCV Ag test kit (AniGen, Seoul, Korea), a fecal swab was mixed with normal saline and preserved in centrifuge tubes, which were packed and mailed to our department and kept in a −80 °C freezer.

### 4.2. Virus Isolation

The cat kidney F81 cell line was used for viral isolation. First, 1 mL of PBS was added to each centrifuge tube containing a fecal sample and oscillated using an oscillator. Then, 500 μL of liquid was drawn and filtered through a 0.22 μm filter (Merck Millipore, Burlington, MA, USA). By digesting cells with 0.05% pancreatin, a cell suspension was prepared and maintained in minimal essential medium (MEM, Gibco, Waltham, MA, USA) supplemented with 10% calf serum. The cell suspension was added to the culture flask and inoculated with CPV-2 at a proportion of 5%. The culture flask was maintained in a CO_2_ incubator (MEMMERT, Schwabach, Germany). We harvested the virus when the cytopathic effect (CPE) was approximately 80%. The third passage of viruses was used for analysis.

### 4.3. Detection of CPV-2

A pair of primers was designed to detect CPV-2. The forward primer was CTGTGGGTAATGTTGGTTGTT (5′ to 3′), and the reverse primer was TGGTCTTGATGTTGATGGATG (5′ to 3′). The expected product length was 1163 bp. We used DNAiso Reagent (TaKaRa, Beijing, China) to extract the viral genome and ExTaq DNA Polymerase (TaKaRa) to amplify the gene. The following amplification procedure was applied: initial denaturation at 94 °C for 5 min, then 35 cycles of denaturation at 94 °C for 30 s, annealing at 48 °C for 30 s and extension at 72 °C for 1 min. The final extension was performed at 72 °C for 7 min.

### 4.4. Amplification and Sequencing of the VP2 Gene

A pair of primers was designed to amplify the whole VP2 gene. The forward primer was CACCAGATCATCCATCAACATC (5′ to 3′), and the reverse primer was AACCACCCACACCATAAC (5′ to 3′). The expected product was 2293 bp in length, encompassing the entire VP2 gene. Super-fidelity DNA Polymerase used with 2 × Phanta Max Master Mix (Vazyme, Nanjing, China). The amplification conditions were established following the manufacturer’s instructions: initial denaturation at 95 °C for 30 s, then 35 cycles of denaturation at 95 °C for 15 s, annealing at 56 °C for 15 s and extension at 72 °C for 1.5 min, and a final extension at 72 °C for 5 min.

The PCR product was purified by an EasyPure Quick Gel Extraction Kit (TransGen, Beijing, China) according to the manufacturer’s instructions. Then, the product was cloned into the pEASY-Blunt cloning vector (TransGen). The recombinant vector was transformed into Trans1-T1 Phage Resistant Chemically Competent Cells (TransGen). Positive clones were screened by blue-white selection and further verified by PCR; finally, they were sent to a third-party company (Huada Gene, Beijing, China) for sequencing.

### 4.5. Sequence Analysis and Phylogenetic Construction

Sequences were assembled using SeqMan (Lasergene, DNASTAR, Madison, WI, USA), and then the nucleotide sequences and deduced amino acid sequences were aligned by the ClustalW method. To construct a nucleotide phylogenetic tree based on the full VP2 gene, MEGA-X was used to find the best DNA models with the maximum likelihood (ML) method. A bootstrap value of 1000 was used to analyze the confidence level. All strains were analyzed with reference strains obtained from the GenBank database. Three CPV-2 vaccine strains and two FPV vaccine strains were included.

### 4.6. Temporal Distribution Analysis

Data on Chinese CPV-2 strains reportedly identified between 2014 and 2019 with clear backgrounds were collected either from GenBank or related publications, and with data on the CPV-2 strains collected specifically for this study (CPV-2 strains collected in 2020 and 2021 were excluded), were subjected to temporal distribution analysis.

## 5. Conclusions

Seven new CPV-2a (297Ala, 426Asn), 21 CPV-2c (426Glu), and 2 FPV strains were identified from canine samples, and CPV-2c emerged as the dominant genotype, which was further proven by the analysis of 683 Chinese strains detected between 2014 and 2019. Currently, no ascertainable facts prove that CPV-2c results in immunization failure. However, the obvious epidemic superiority of CPV-2c strains with VP2 capsid mutations at Ala5Gly and Gln370Arg in Asian areas may pose a new challenge to the effectiveness of currently used vaccines. Further research should be performed to investigate the relationship between immunization failure and CPV-2c strains. The phylogenetic tree based on the VP2 gene revealed the national and international spread of CPV-2 variants; thus, boundary administration should be stricter. One unanticipated finding was the identification of two FPV strains in canine samples, which is an important finding that warrants future research.

## Figures and Tables

**Table 1:** Information of canine parvovirus (CPV-2) strains in this study.

Strain	Collected Time	Collected Area	Source	Genotype	Genbank Accession Number
CPV2018/3	2018.1	Guangdong	Cell culture	CPV-2c	MW182694
CPV2018/4	2018.1	Guangdong	Cell culture	New CPV-2a	MW182695
CPV2018/5	2018.1	Guangdong	Cell culture	New CPV-2a	MW182696
CPV2018/6	2018.1	Tianjin	Cell culture	New CPV-2a	MW691123
CPV2018/9	2018.11	Guangdong	Cell culture	CPV-2c	MW182697
CPV2018/10	2018.11	Guangdong	Cell culture	CPV-2c	MW182698
CPV2018/11	2018.12	Tianjin	Fecal sample	CPV-2c	MW182713
CPV2018/12	2018.12	Tianjin	Cell culture	New CPV-2a	MW182699
CPV2018/13	2018.12	Tianjin	Cell culture	CPV-2c	MW182700
CPV2018/14	2018.12	Tianjin	Cell culture	CPV-2c	MW182711
CPV2018/15	2018.12	Tianjin	Fecal sample	CPV-2c	MW182714
CPV2018/16	2018.12	Tianjin	Cell culture	New CPV-2a	MW182702
CPV2018/17	2018.12	Tianjin	Fecal sample	CPV-2c	MW182715
CPV2018/18	2018.12	Tianjin	Cell culture	New CPV-2a	MW182716
CPV2018/19	2018.12	Tianjin	Cell culture	CPV-2c	MW182701
CPV2018/20	2018.12	Guangdong	Cell culture	CPV-2c	MW182703
CPV2018/22	2018.12	Guangdong	Cell culture	CPV-2c	MW182712
CPV2019/1	2019.1	Tianjin	Cell culture	CPV-2c	MW182704
CPV2019/2	2019.1	Tianjin	Cell culture	CPV-2c	MW182705
CPV2019/3	2019.2	Tianjin	Cell culture	CPV-2c	MW182717
CPV2019/4	2019.2	Tianjin	Fecal sample	CPV-2c	MW182709
CPV2019/5	2019.2	Tianjin	Cell culture	CPV-2c	MW182706
CPV2019/6	2019.2	Tianjin	Cell culture	New CPV-2a	MW182718
CPV2019/7	2019.2	Tianjin	Cell culture	CPV-2c	MW182710
CPV2020/1	2020.5	Beijing	Cell culture	FPV	MW182707
CPV2020/2	2020.5	Beijing	Cell culture	FPV	MW182708
CPV2020/3	2020.6	Henan	Cell culture	CPV-2c	MW182719
CPV2020/5	2020.8	Henan	Fecal sample	CPV-2c	MW182720
CPV2020/6	2020.1	Henan	Cell culture	CPV-2c	MW691124
CPV2021/1	2021.1	Tianjin	Fecal sample	CPV-2c	MW691122

**Table 2:** Amino acid variations in the VP2 region of CPV-2 strains in this study compared with reference strain.

Stain	Amino Acid Variation Site in the VP2 Protein																					
	5	13	80	87	93	101	103	232	238	267	297	300	305	323	324	370	375	426	440	564	568	570
M38245 CPV-2	Ala	Pro	Arg	Met	Asn	Ile	Ala	Ile	Pro	Phe	Ser	Ala	Asp	Asn	Tyr	Gln	Asn	Asn	Thr	Ser	Gly	Lys
CPV2018/3	Gly	.	.	Leu	.	Thr	.	.	.	Tyr	Ala	Gly	Tyr	.	Ile	Arg	Asp	Glu	.	.	.	.
CPV2018/4	.	.	.	Leu	.	Thr	.	.	.	Tyr	Ala	Gly	Tyr	.	Ile	.	Asp	.	Ala	.	.	.
CPV2018/5	.	.	.	Leu	.	Thr	.	.	.	Tyr	Ala	Gly	Tyr	.	Ile	.	Asp	.	Ala	.	.	.
CPV2018/6	.	.	.	Leu	.	Thr	.	.	.	Tyr	Ala	Gly	Tyr	.	Ile	.	Asp	.	Ala	.	.	.
CPV2018/9	Gly	.	.	Leu	.	Thr	.	.	.	Tyr	Ala	Gly	Tyr	.	Ile	Arg	Asp	Glu	.	.	.	Arg
CPV2018/10	Gly	.	.	Leu	.	Thr	.	.	.	Tyr	Ala	Gly	Tyr	.	Ile	Arg	Asp	Glu	.	.	.	.
CPV2018/11	Gly	.	.	Leu	.	Thr	.	.	.	Tyr	Ala	Gly	Tyr	.	Ile	Arg	Asp	Glu	.	.	.	.
CPV2018/12	.	.	.	Leu	.	Thr	.	.	.	Tyr	Ala	Gly	Tyr	.	Ile	.	Asp	.	Ala	.	.	.
CPV2018/13	Gly	.	.	Leu	.	Thr	.	.	.	Tyr	Ala	Gly	Tyr	.	Ile	Arg	Asp	Glu	.	.	.	.
CPV2018/14	Gly	.	.	Leu	.	Thr	.	.	.	Tyr	Ala	Gly	Tyr	.	Ile	.	Asp	Glu	.	.	.	.
CPV2018/15	Gly	.	.	Leu	.	Thr	.	.	.	Tyr	Ala	Gly	Tyr	.	Ile	Arg	Asp	Glu	.	.	.	.
CPV2018/16	.	.	.	Leu	.	Thr	.	.	.	Tyr	Ala	Gly	Tyr	.	Ile	.	Asp	.	Ala	.	.	.
CPV2018/17	Gly	.	.	Leu	.	Thr	.	.	.	Tyr	Ala	Gly	Tyr	.	Ile	Arg	Asp	Glu	.	.	.	.
CPV2018/18	.	Ser	.	Leu	.	Thr	.	.	.	Tyr	Ala	Gly	Tyr	.	Ile	.	Asp	.	Ala	.	.	.
CPV2018/19	Gly	Ser	.	Leu	.	Thr	.	.	.	Tyr	Ala	Gly	Tyr	.	Ile	Arg	Asp	Glu	.	.	.	.
CPV2018/20	Gly	Ser	.	Leu	.	Thr	.	.	.	Tyr	Ala	Gly	Tyr	.	Ile	Arg	Asp	Glu	.	.	.	.
CPV2018/22	Gly	.	.	Leu	.	Thr	.	.	.	Tyr	Ala	Gly	Tyr	.	Ile	Arg	Asp	Glu	.	.	.	.
CPV2019/1	Gly	.	.	Leu	.	Thr	.	.	.	Tyr	Ala	Gly	Tyr	.	Ile	Arg	Asp	Glu	.	.	.	.
CPV2019/2	Gly	.	.	Leu	.	Thr	.	.	.	Tyr	Ala	Gly	Tyr	.	Ile	Arg	Asp	Glu	.	.	.	.
CPV2019/3	Gly	.	.	Leu	.	Thr	.	.	.	Tyr	Ala	Gly	Tyr	.	Ile	.	Asp	Glu	.	.	.	.
CPV2019/4	Gly	.	.	Leu	.	Thr	.	.	.	Tyr	Ala	Gly	Tyr	.	Ile	Arg	Asp	Glu	.	.	.	.
CPV2019/5	Gly	.	.	Leu	.	Thr	.	.	.	Tyr	Ala	Gly	Tyr	.	Ile	Arg	Asp	Glu	.	.	.	.
CPV2019/6	Gly	.	.	Leu	.	Thr	.	.	.	Tyr	Ala	Gly	Tyr	.	Ile	.	Asp	.	Ala	.	.	.
CPV2019/7	Gly	.	.	Leu	.	Thr	.	.	.	Tyr	Ala	Gly	Tyr	.	Ile	Arg	Asp	Glu	.	.	.	.
CPV2020/1	.	.	Lys	.	Lys	Thr	Val	Val	Gln	.	.	.	.	Asp	.	.	Asp	.	.	Asn	Ala	.
CPV2020/2	.	.	Lys	.	Lys	Thr	Val	Val	.	.	.	.	.	Asp	.	.	Asp	.	.	Asn	Ala	.
CPV2020/3	Gly	.	.	Leu	.	Thr	.	.	.	Tyr	Ala	Gly	Tyr	.	Ile	Arg	Asp	Glu	.	.	.	.
CPV2020/5	Gly	.	.	Leu	.	Thr	.	.	.	Tyr	Ala	Gly	Tyr	.	Ile	Arg	Asp	Glu	.	.	.	.
CPV2020/6	Gly	.	.	Leu	.	Thr	.	.	.	Tyr	Ala	Gly	Tyr	.	Ile	Arg	Asp	Glu	.	.	.	.
CPV2021/1	Gly	.	.	Leu	.	Thr	.	.	.	Tyr	Ala	Gly	Tyr	.	Ile	Arg	Asp	Glu	.	.	.	.

## Data Availability

The data presented in this study are contained within the article.

## Supplementary Materials

The following are available online at https://www.mdpi.com/article/10.3390/pathogens10050588/s1, Table S1: The data of 683 Chinese CPV-2 strains.
